# Monocarboxylate Transporter 8 Deficiency: From Pathophysiological Understanding to Therapy Development

**DOI:** 10.3389/fendo.2021.723750

**Published:** 2021-09-01

**Authors:** Ferdy S. van Geest, Nilhan Gunhanlar, Stefan Groeneweg, W. Edward Visser

**Affiliations:** Academic Center For Thyroid Disease, Department of Internal Medicine, Erasmus Medical Center, Rotterdam, Netherlands

**Keywords:** MCT8 deficiency, monocarboxylate transporter 8, Allan-Herndon-Dudley syndrome (AHDS), thyroid hormone transport, thyroid hormone signaling

## Abstract

Genetic defects in the thyroid hormone transporter monocarboxylate transporter 8 (MCT8) result in MCT8 deficiency. This disorder is characterized by a combination of severe intellectual and motor disability, caused by decreased cerebral thyroid hormone signalling, and a chronic thyrotoxic state in peripheral tissues, caused by exposure to elevated serum T3 concentrations. In particular, MCT8 plays a crucial role in the transport of thyroid hormone across the blood-brain-barrier. The life expectancy of patients with MCT8 deficiency is strongly impaired. Absence of head control and being underweight at a young age, which are considered proxies of the severity of the neurocognitive and peripheral phenotype, respectively, are associated with higher mortality rate. The thyroid hormone analogue triiodothyroacetic acid is able to effectively and safely ameliorate the peripheral thyrotoxicosis; its effect on the neurocognitive phenotype is currently under investigation. Other possible therapies are at a pre-clinical stage. This review provides an overview of the current understanding of the physiological role of MCT8 and the pathophysiology, key clinical characteristics and developing treatment options for MCT8 deficiency.

## Introduction

Throughout life, thyroid hormone plays an indispensable role in many processes in almost all tissues of the human body. During prenatal and early postnatal life, adequate thyroid hormone signalling is crucial for normal neurodevelopment (1). Furthermore, thyroid hormone regulates key metabolic processes (e.g. mitochondrial respiration) in various tissues, including the liver, kidneys and muscles (2, 3).

Thyroid hormone is the common name for both the prohormone thyroxine (T4), the major product of the thyroid, and the biologically active triiodothyronine (T3). Intracellular thyroid hormone signalling is governed by three major processes: 1) transport of thyroid hormone across the cell membrane, facilitated by specific thyroid hormone transporter proteins, 2) conversion of T4 into T3 or the inactive metabolite reverse (r)T3 and further degradation into other inactive thyroid hormone metabolites by deiodinating enzymes types 1-3 (DIO1-3), and 3) genomic action of T3 upon binding to thyroid hormone receptor (TR) α and β (4). Together, these mechanisms allow for a precise and tissue-specific regulation of intracellular thyroid hormone signalling. This is pivotal for proper development and function of many tissues and crucial for the overall homeostasis of the hypothalamus-pituitary-thyroid (HPT) axis (2, 3). Hence, alterations in any of these mechanisms can result in tissue-specific thyroid hormone signalling defects. In human, defects in all these mechanisms have been identified and such disorders generally impair cellular thyroid hormone signalling (5–12).

**Figure 1:**
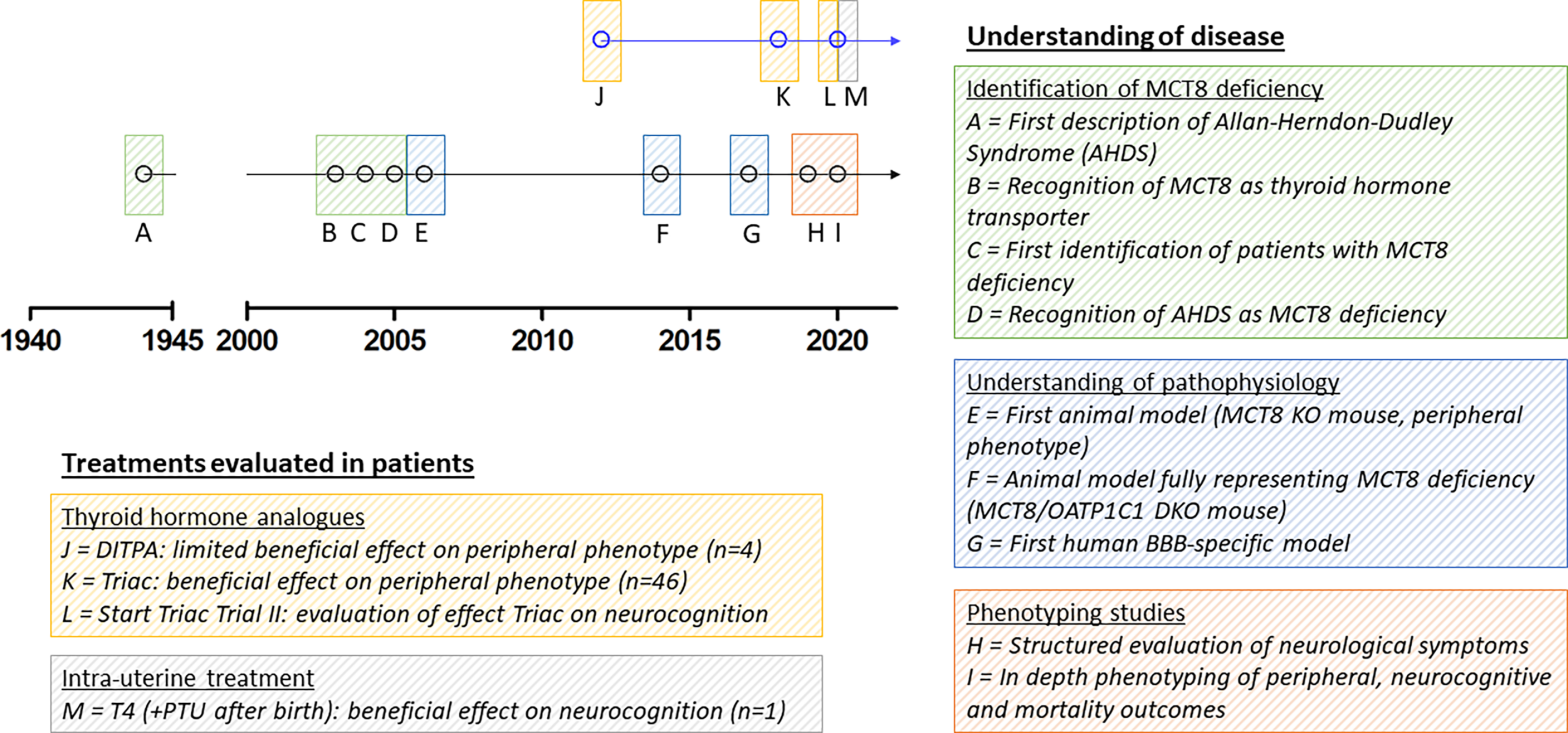
Schematic representation of seminal discoveries in understanding of disease and treatment effects in patients. Due to limited space, we were unable to include all other relevant discoveries on MCT8 deficiency in the figure. MCT8, monocarboxylate transporter 8; AHDS, Allan-Herndon-Dudley Syndrome; KO, knock-out; OATP1C1, organic anion transporter 1C1; DKO, double knock-out; BBB, blood-brain barrier; DITPA, diiodothyropropionic acid; T4, thyroxine; PTU, propylthiouracil.

To date, the most specific thyroid hormone transporter identified is monocarboxylate transporter (MCT) 8, encoded by *SLC16A2* on chromosome Xq13.2 (13). Pathogenic variants in this gene result in a clinical syndrome of severe intellectual and motor disability and increased serum T3 concentrations, leading to thyrotoxic symptoms in peripheral tissues, together known as MCT8 deficiency [also known as Allan-Herndon-Dudley Syndrome (AHDS); OMIM number 300523] (8, 9, 14). Patients generally have poor head control, remain non-verbal, are wheelchair bound and severely underweight and often die at a young age (15). Since the identification of the first series of patients with MCT8 deficiency, many efforts have been undertaken to better understand this rare disorder and to develop potential therapeutic strategies (**Figure 1**). However, important pathophysiological questions remain unanswered to date and treatment options are limited.

## Physiological Role of MCT8

The *SLC16A2* gene comprises two transcriptional start sites, resulting either in an MCT8 protein of 613 amino acids (referred to as the ‘long’ isoform) or 539 amino acids (referred to as the ‘short’ isoform). Although the functional properties of both isoforms are highly similar, the short isoform is generally considered physiologically relevant, since this is the only isoform identified in human tissue to date. *In vitro* overexpression studies have demonstrated a role for the extended N-terminus, specific to the long MCT8 isoform, in ubiquitin-dependent proteasomal degradation, thereby potentially regulating expression of MCT8 protein (16, 17). Yet, there is no definitive proof for the existence of a long MCT8 protein isoform under physiological conditions, which led to the recent change of the reference sequence from the long isoform (NM_006517.3; NP_006508.1) to the short isoform (NM_006517.5; NP_006508.2). It should be emphasized that most literature on MCT8 available to date used the long translational isoform to assign the position of variants. To avoid confusion amongst researchers and clinicians, we recently proposed to continue using the long isoform of MCT8 (counting from the first translational start site) in the nomenclature of *SLC16A2* variants, according to the majority of variants described in literature (18, 19).

MCT8 is capable of mediating the flux of thyroid hormone across the cell membrane through facilitated diffusion, independent of pH or a Na^+^ gradient (18). Major substrates of MCT8 are the iodothyronines T3 and T4; to a lesser extent, it is also capable to mediate transport of the inactive metabolites rT3 and 3,3-diiodothyronine (3,3’-T2) (20). Both uptake and efflux of thyroid hormone across the cell membrane are facilitated by MCT8 (21). Interestingly, transport of thyroid hormone metabolites lacking the αNH2 group [e.g. 3,3’,5-triiodothyroacetic acid (Triac) and 3,3′,5,5′-tetraiodothyroacetic acid (Tetrac)] is not dependent on MCT8 (22, 23). Silychristin, a flavonolignan found in some traditional European and Asian medicinal compounds, is capable of specifically and effectively blocking MCT8-mediated thyroid hormone transport in different *in vitro* and *ex vivo* models (24, 25). Based on studies using the inhibitors bromsulphthalein (BSP) and desipramine, Jomura et al. suggest that MCT8 can be involved in efflux of the anti-epileptic drug phenytoin across the blood-brain barrier (BBB) (26). However, as these inhibitors are not MCT8-specific and with BSP being an inhibitor of the majority of thyroid hormone transporting organic anion transporter proteins (OATPs) (18), additional evidence is warranted to support this hypothesis.

MCT8 is ubiquitously expressed throughout the human body. Both *MCT8* mRNA and protein are most prominently expressed in the liver, but are also found in significant quantities in the thyroid, kidneys, pituitary and brain (27). In particular, MCT8 is expressed in different neural cells, including subtypes of neurons, astrocytes, oligodendrocytes and tanycytes (28, 29). Expression of MCT8 in tanycytes may potentially play an important role in the negative feedback of HPT axis homeostasis (30). During all stages of intra-uterine development, MCT8 expression is observed in vascular structures within the foetal brain and, later in development, in their surrounding astrocytes (31). Moreover, MCT8 expression is observed in radial glial cells, leptomeningeal cells and blood vessels in the subarachnoid space of murine models (at 14 to 38 gestational weeks) (31). These findings are indicative of a prominent role for MCT8 at the BBB and, to a lesser extent, also at the blood-cerebrospinal fluid barrier (BCSFB), and underlie the current paradigm that MCT8 is crucial for thyroid hormone transport across the BBB in particular. Interestingly, recent detailed protein expression studies from Wilpert et al. on murine brain tissues demonstrated strong expression of MCT8 in the brain barriers and many subpopulations of neurons (including cortical and cerebellar neurons) at a young age (postnatal day 6, representative for the foetal phase in human), but a sharp decrease in MCT8 expression in neurons upon aging, whereas expression in the BBB and BCSFB did not change upon aging. In adult post-mortem and fresh human brain tissues from 4 older individuals (50 – 82 years), a similar pattern of strong MCT8 expression in endothelial cells and minimal to no expression in neuronal tissues was found (32). Together, these findings indicate the presence of MCT8 in the majority of brain tissues during (prenatal) development, whereas its presence may be restricted to the brain barriers later in adult life. Yet, it is hitherto unknown how MCT8 functionally contributes to intracellular thyroid hormone homeostasis in different cell types at different developmental stages.

Despite its ubiquitous expression in human tissues other than brain (so called peripheral tissues), the physiological role of MCT8 in these tissues is less well-defined. Importantly, along with MCT8, various alternative thyroid hormone transporters are ubiquitously expressed throughout the peripheral tissues (members of the L-type amino acid transporter and OATP family, as well as Na+-taurocholate cotransporting polypeptide and the recently identified thyroid hormone transporter SLC17A4) (18). Hence, other peripheral tissues have a variable dependence on MCT8 for adequate thyroid hormone homeostasis (33, 34).

MCT8 is highly expressed on the basolateral side of follicular epithelial cells of the murine thyroid, as demonstrated by different *in situ* hybridization, immunohistochemistry and immunoblot analyses (35, 36). Similar observations were made in the human thyroid (37).

## Pathophysiology of MCT8 Deficiency

The relevance of adequate MCT8-mediated thyroid hormone transport becomes apparent in patients harbouring mutations in the *SLC16A2* gene. Such mutations result in a phenotype of severe intellectual and motor disability and signs of peripheral thyrotoxicosis, including negative clinical sequelae as tachycardia and being severely underweight. As *SLC16A2* is located on the X chromosome, mostly men are affected; one female with MCT8 deficiency due to unfavourable X-inactivation has been described (38). A detailed description of the clinical characteristics of this disease is provided in section *Disease Characteristics* of this review.

Different animal models have been exploited to better understand the pathophysiology of MCT8 deficiency. The first animal model generated for these purposes was the Mct8 knockout (KO) mouse (39, 40). In mice, which are commonly studied to delineate tissue-specific intracellular thyroid hormone signalling processes, the expression pattern of MCT8 is largely comparable to the human situation (18). However, in contrast to the human BBB, the BBB of mice (and other rodents) co-expresses the T4-specific transporter Oatp1c1, enabling T4 transport into the brain and subsequent local conversion into T3 by Dio2 (41). This observation is highly relevant in studying cerebral thyroid hormone homeostasis, as murine models do not well resemble the human physiological situation. Although Mct8 KO mice well resemble the serum thyroid hormone fingerprint of patients with MCT8 deficiency, no overt neurological abnormalities were identified. Therefore, results obtained in rodent brains should be translated with caution to the human situation (42). Hence, Mct8 KO mice are generally considered to be an adequate model for the peripheral thyrotoxic state observed in patients with MCT8 deficiency, but should not be used when evaluating (treatment effects on) the neurocognitive phenotype (42). Subsequently, an Mct8/Oatp1c1 double KO (DKO) mouse model was generated, which indeed mimicked both the peripheral thyrotoxic as well as the neurocognitive phenotype of MCT8 deficiency. The latter was illustrated by pronounced locomotor abnormalities, altered Purkinje cell dendritogenesis, a reduction in cerebral T3 and T4 content to 10%, decreased cerebral expression of the thyroid hormone sensitive gene *Hairless* and reduced cerebral levels of myelin basic protein (MBP), indicating hypomyelination (41). More recent studies suggest that Mct8/Dio2 DKO mice may also mimic human MCT8 deficiency, as inactivation of Dio2 prevents the formation of sufficient intracerebral T3 concentrations even in presence of functional Oatp1c1 (43). In addition to murine models, zebrafish and chicken models are available and resemble parts of the phenotypic spectrum of MCT8 deficiency (44, 45).

The functional contribution of MCT8 in the BBB only became recently apparent when Vatine et al. successfully obtained MCT8 deficient vascular endothelial and neural cells from patient-derived induced pluripotent stem cells (iPSCs) (46). In these MCT8 deficient neural cells, reduced thyroid hormone uptake capacity was found. However, the ability of these MCT8 deficient iPSCs to differentiate to neural cells was grossly unaltered. This latter observation suggests that, despite the absence of functional MCT8, sufficient intracellular T3 concentrations are reached in these cells. Utilizing iPSCs to model the BBB demonstrated the relevance of MCT8 in this barrier, with transport of T3 across the modelled BBB being significantly reduced in absence of MCT8 (46). Defective MCT8 in specific neuronal populations or in neural stem cells from patient derived iPSCs was not studied. Complementary studies of Mayerl et al. on adult hippocampal neurogenesis in murine neural stem cells showed that this process is largely regulated by thyroid hormone signalling in a cell-autonomous fashion, with MCT8 functioning as an important gate keeper (47). Upon both global deletion and adult neural stem cell-specific deletion of MCT8, differentiation of hippocampal neuroblasts was reduced. Similarly, recent studies of Vatine et al. demonstrated that oligodendrocyte precursor cells (OPCs) are not able to generate myelinating oligodendrocytes in the context of reduced cerebral thyroid hormone signalling, as observed in MCT8 deficiency (29). It should be noted that these studies used different models representing different neural cell types and different stages of neuronal development, limiting direct comparison of their results. López-Espíndola et al. performed post-mortem studies on the cerebral cortex and cerebellum of a human foetus with MCT8 deficiency (30 gestation weeks) and an 11-year old patient with MCT8 deficiency (48). The foetal brain already showed delayed cortical and cerebellar development (including altered Purkinje cell dendritogenesis), hypomyelination (indicated by low levels of MBP) and impaired maturation of the axons, indicating that the brain suffers from a severe state of low thyroid hormone signalling during pregnancy. Similar features were identified in the brain of the 11-year old patient, thus excluding spontaneous recovery of the neural aberrations with increasing age. Cerebral cortex T3 and T4 concentrations were reduced by 50%. The observed features were in line with the features found in Mct8/Oatp1c1 DKO mice (41). Interestingly, the reduction in cerebral thyroid hormone content was less prominent when compared to Mct8/Oatp1c1 DKO mice. This remarkable difference is currently not well understood; amongst other factors, differential expression of so far unidentified thyroid hormone transporters or other protecting mechanisms could underlie this observation. Together with the studies of Wilpert et al., these studies point towards a prominent role of MCT8 in the transport of thyroid hormone across the brain barriers, and the role of MCT8 in neural cells appears to vary between cell types and developmental stages.

**Figure 2:**
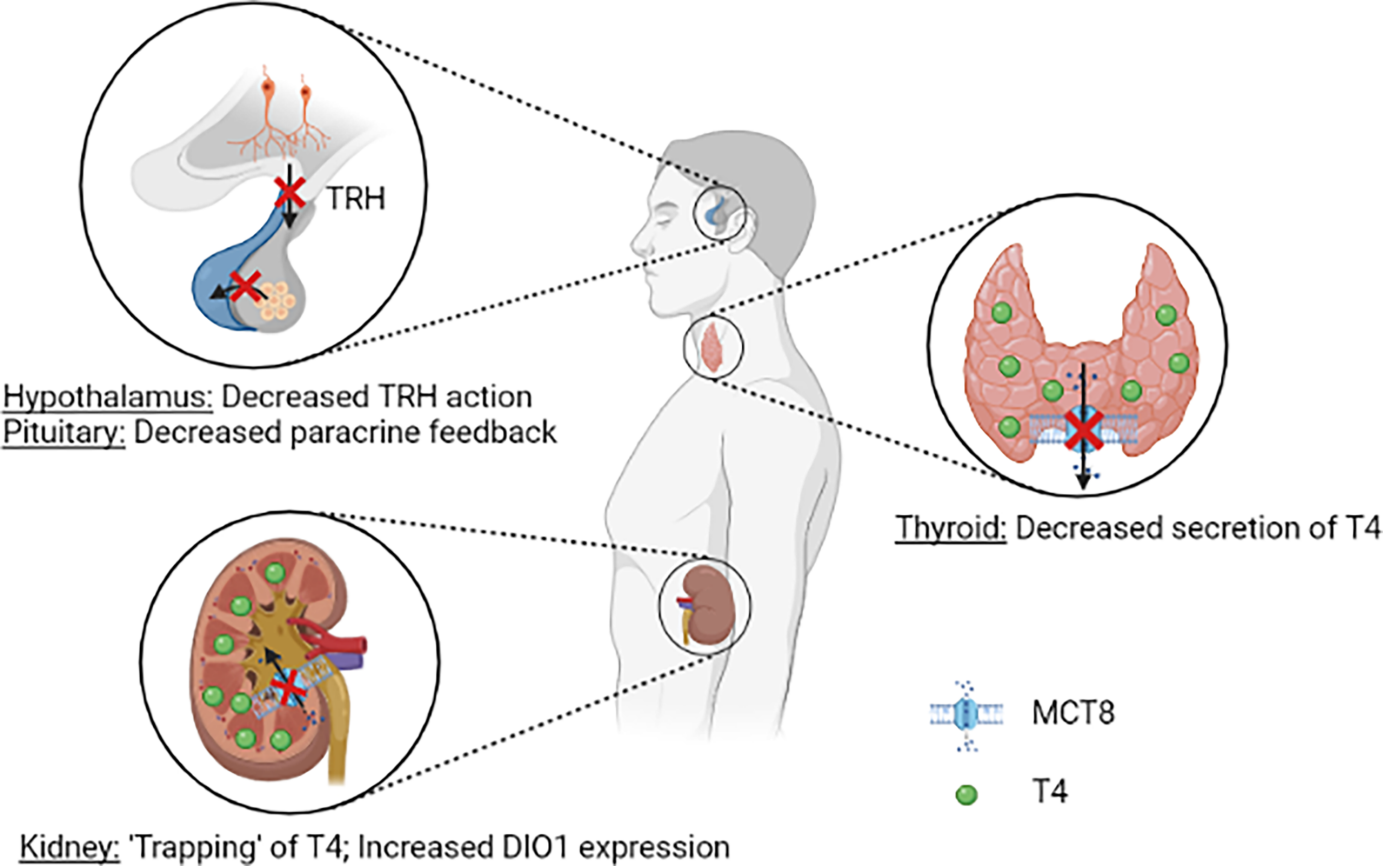
Schematic representation of the current understanding of potential pathogenic mechanisms underlying the abnormal serum thyroid hormone concentrations in patients MCT8 deficiency. Loss of MCT8 function may lead to altered thyroid hormone sensitivity in the hypothalamus and pituitary (upper left panel). Loss of MCT8 function results in decreased thyroidal T4 secretion (right panel). Loss of MCT8 function may results in decreased excretion and ‘trapping’ of T4 in the kidneys, and subsequent increase in DIO1 expression. TRH, thyrotropin releasing hormone; T4, thyroxine; MCT8, monocarboxylate transporter 8; DIO1, deiodinase type 1.

Over the years, multiple hypotheses have been postulated on the pathophysiology of the distinct thyroid hormone fingerprint in MCT8 deficiency. It was first reasoned that, due to reduced cerebral T3 uptake, a progressive buildup of T3 in serum stimulates DIO1 activity in peripheral tissues, thus further aggravating the peripheral thyrotoxicosis by enhancing T4 to T3 conversion. In Mct8/Pax8 DKO and Pax8 KO mice, both being completely athyroid, injection of T3 resulted in similar serum T3 concentrations in both groups (35). In contrast, injection of T4 resulted in lower serum T4 concentrations in Mct8/Pax8 DKO mice compared to Pax8 KO mice, indicating increased deiodinase activity independent of the circulating T3 concentrations. Hence, a reduced cerebral thyroid hormone uptake does not explain the characteristic high T3:T4 ratio observed in MCT8 deficiency. As MCT8 is expressed in human tanycytes and thyrotropin releasing hormone (TRH)-expressing neurons (28, 49), it was hypothesized that MCT8 may have an important role in controlling the HPT axis, and in particular the negative feedback system. Moreover, the co-expression of MCT8 and DIO2 in folliculostellate cells of the human pituitary indicates that MCT8 might also control the HPT axis on pituitary level, with folliculostellate cells acting in a paracrine fashion (49). In Mct8 KO mice the thyroidal secretion of T4 is decreased, whereas increased release of T3 is observed (35, 36). It should be noted that the ratio of thyroidal T3 *vs* T4 secretion and intrathyroidal deiodinase activity are different between rodents and humans (50). Taken together, it is likely that MCT8 plays a role in thyroid hormone homeostasis at all levels of the HPT axis (**Figure 2**). This, however, does not completely explain the high serum T3:T4 ratio that is observed in all patients with MCT8 deficiency (see *Disease Characteristics*), as the increased peripheral thyroid hormone metabolism remains not well understood in this hypothesis.

A third hypothesis focusses on deiodinase activity in peripheral tissues. Dio1 has a role in rT3 clearance and converts the prohormone T4 into biologically active T3. Dio2 has an activating function by catalysing T4 to T3 conversion, whereas Dio3 primarily has a role in inactivation of thyroid hormone (conversion of T4 to rT3 and T3 to T2) (4). In the liver and kidneys of Mct8 KO mice, strongly increased activity of Dio1 was observed, whereas increased Dio2 activity was found in the brain, pituitary and skeletal muscles (39, 40). In these mice, increased liver T3 content and increased expression of Dio1 were observed, and resulted in clinical features of increased thyroid hormone signalling in the liver (e.g. decreased serum cholesterol concentrations), in line with observations made in patients with MCT8 deficiency (15, 39). By utilizing Mct8/Dio1 DKO mice, Liao et al. demonstrated that the alterations in Dio1 activity are primarily responsible for the high T3:T4 ratio in Mct8 KO mice (51). Interestingly, proximal tubule cells that normally express MCT8, showed increased Dio1 expression in the absence of MCT8. Moreover, upon peripheral injection of radiolabelled T3 and T4 in Mct8 KO mice, increased accumulation of radioactivity was observed in kidney, but not in liver homogenates (40). Also, liver-specific KO of Dio1 in Mct8 KO mice did not result in normalization of the circulating thyroid hormone levels, suggesting that the increased hepatic Dio1 expression is not causing the abnormal thyroid hormone levels (52). Together, these findings hint towards a crucial role for MCT8 in renal T4 efflux, thereby maintaining global thyroid hormone homeostasis (**Figure 2**). Based on these findings, it is currently hypothesized that, due to deficient MCT8-mediated thyroid hormone excretion, T4 is trapped in the kidneys, which results in an upregulation of Dio1. Subsequent to this increased expression, T4 is rapidly conversed to T3 by Dio1 and released to the circulation by other transporters, resulting in increased serum T3 concentrations. However, further studies are warranted to confirm if, and decipher how the kidneys contribute to the distinct fingerprint of thyroid function tests in MCT8 deficiency.

## Disease Characteristics

In 1944, Allan, Herndon and Dudley first described families with several members suffering from an X-linked form of neurodevelopmental delay, resulting in a lack of speech and impaired walking ability. Hence, it was coined Allan-Herndon-Dudley Syndrome or AHDS (53). The underlying pathogenic mechanism of AHDS remained unclear up until 2004. Soon after the identification of MCT8 as a specific thyroid hormone transporter (13), the first patients with a mutation therein were identified, presenting a remarkably similar clinical phenotype as those males described by Allan, Herndon and Dudley (8, 9). In 2005, Schwartz et al. provided definite proof that the families originally described by Allan, Herndon and Dudley indeed carried mutations in MCT8 (14). Thus, the terms MCT8 deficiency and AHDS both comprise the same syndrome and are both commonly used in the field. With the current tendency to avoid eponyms, MCT8 deficiency is currently preferred to describe the disease.

Initial reports mainly focused on the neurodevelopmental phenotype, resulting from defective thyroid hormone transport into the brain. Over time, it was increasingly recognized that, in contrast to the brain, other tissues of the human body (hereafter called peripheral tissues) are in a chronic thyrotoxic state. Due to the abundance of alternative thyroid hormone transporters in peripheral tissues, these tissues adequately sense the high circulating T3 concentrations.

When studying the genetic basis of MCT8 deficiency, it is critical to discriminate deleterious mutations from benign (rare) variants (54). Approximately 150 different mutations in about 250 different families have been described in literature and a prevalence of 1:70,000 males has been suggested (15, 55). They can be classified in three major groups: large deletions resulting in an incomplete MCT8 protein, insertions/deletions/nonsense mutations resulting in a frameshift or premature truncation, and missense mutations resulting in a single amino acid change (18). Whereas the pathogenic character of the first two categories can be predicted with considerable certainty, not all missense variants impair MCT8 thyroid hormone transport function. Evaluation of residual thyroid hormone transport capacity of these variants in *in vitro* systems or patient-derived fibroblasts is therefore indispensable to confirm the pathogenic nature of identified variants (56–58). Recent studies suggested that C-terminal missense variants beyond amino acid residue Met574 (long isoform) are well-tolerated and likely to not results in a phenotype (59). Additional studies are warranted to further delineate the relationship between the different mutations and the phenotypic variability.

In the years following its discovery, understanding of the phenotype of MCT8 deficiency largely relied on reports of individual cases and studies including small cohort of patients with MCT8 deficiency. With the number of identified patients strongly increasing with rising awareness amongst physicians in recent years, the opportunity arose to study larger cohorts of patients (15, 60). Following structured evaluation of two cohorts, better understanding of different aspects of MCT8 deficiency was obtained. The first cohort, described by Remerand et al., comprised of 24 patients in whom specifically the neurological features were further detailed (60). In line with previously reports, they showed that the neurocognitive phenotype of MCT8 deficiency comprises severe intellectual and motor disability. Hypotonia, spasticity and dystonia are key clinical features (observed in 100%, 71% and 75%, respectively). The majority of patients had poor head control, did not develop speech and did not attain the ability to sit or walk. Magnetic resonance imaging (MRI) depicted hypomyelination in the majority of patients (19 out of 24 patients) and global brain atrophy in approximately 50% of patients. Similar observations were made earlier, in 13 MRI-scans of 6 patients (61). Moreover, detailed diffusion tensor imaging, available in three subjects, demonstrated a lack of definition in the anteroposteriorly directed association tracts, whereas the commissural white matter tracts (indicating the corpus callosum) and the corticospinal tracts appeared relatively normal. A recent review of the available literature suggested that MRI-scans of patients show gradual improvement of myelination throughout life, indicating a state of delayed myelination rather than hypomyelination (62). In contrast, post-mortem microscopic evaluation of the brain of an 11-year old patient demonstrated deficient myelination [also see *Pathophysiology of MCT8 Deficiency* (48)]. Strikingly, in the cohort of Remerand and colleagues, 8 out of 24 (33%) patients were able to walk, indicative of a less severe phenotype. This proportion was considerably higher than estimates based on available case reports and case series (18, 61), suggesting that patients with a relatively less severe phenotype were overrepresented in this cohort. Although Remerand et al. reported on thyroid function tests, the peripheral phenotype had not been further detailed.

Hence, the establishment of a larger cohort was warranted, in order to obtain more robust phenotypic data covering both the neurological and peripheral phenotype. In an international multicenter effort, Groeneweg et al., described the phenotypic characteristics of up to 151 patients with MCT8 deficiency (15). Similar observations of key clinical neurological symptoms were found as in the cohort of Remerand et al., albeit with a much lower proportion of patient who had developed walking abilities (4 out of 77 patients). Also, this study was the first to systematically collect quantitative data on the motor and cognitive abilities of patients with MCT8 deficiency, as assessed with the Gross Motor Function Measure-88 and Bayley Scales of Infant Development III. These abilities plateaued at a median developmental age of well below 12 months, whereas the median age of evaluation was 6.4 years.

**Figure 3:**
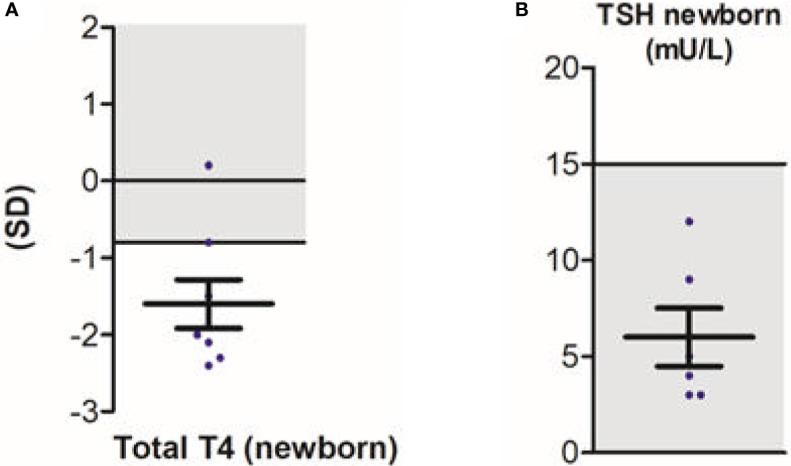
Neonatal screening in patients with MCT8 deficiency; total T4 **(A)** and TSH **(B)**. Blue dots represent individual data points. Black bars indicate the mean ± SD. Grey areas indicate reference intervals. This figure was published in Disease characteristics of MCT8 deficiency: an international, retrospective, multicentre cohort study, Lancet Diabetes Endocrinol. 8(7), S Groeneweg et al., Figures 3D and s9I, 594-605, Copyright Elsevier (2020).

Next to neurodevelopmental symptoms, this study was the first to provide large-scale data on the peripheral phenotype (15). Observed hallmark features were elevated serum T3 concentrations (present in 95% of patients), hypothyroxinaemia (present in 89% and 90% of patients for serum free T4 and total T4 concentrations, respectively), with serum thyroid stimulating hormone (TSH) concentrations within the age-specific reference range in the majority (89%) of patients. Serum rT3 concentrations were low in 91% of patients, resulting in a high T3:rT3 ratio in all patients. No difference in serum T3 concentrations was observed in patients with severe *vs* less severe phenotype. In contrast, patients with a less severe phenotype showed significantly higher total T4 concentrations when compared to patients with a severe phenotype (although still below the reference interval). As a consequence, the T3:T4 ratio was lower in patients with a less severe phenotype. Importantly, of eight patients with T4-based neonatal screening data available, the majority of patients (88%) had total T4 concentrations below the 20^th^ percentile (**Figure 3A**). None were identified through neonatal screening. Also, none would be identified as abnormal in TSH-based screening programs, which are commonly utilized (**Figure 3B**). Recent studies demonstrated that rT3 concentrations and T3/rT3 ratio might be suitable biomarkers in neonatal screening (63). As pregnancy and delivery are unremarkable in most cases (15) and treatment early in life can potentially improve the neurocognitive phenotype, early identification of patients with MCT8 deficiency through neonatal screening could be of large additive value.

As a consequence of the elevated serum T3 concentrations, clinical parameters and biochemical markers reflecting thyroid hormone action in peripheral tissues were altered. Being underweight was detected in 71% of patients and 84% of patients had hypotrophic musculature. Recurrent pulmonary infections were common, which might be related to the high incidence of impaired swallowing function (observed in 71% of patients) and increased susceptibility as a result of being underweight. Moreover, signs of cardiovascular dysfunction were commonly observed, including premature atrial contractions (PACs), resting tachycardia and elevated systolic blood pressure (in 76%, 31% and 53% of patients, respectively). Serum sex hormone-binding globulin (SHBG) concentrations, considered to be a marker of thyroid hormone signalling in the liver, were elevated compared to its age-specific reference intervals in 88% of patients.

Whereas life span had previously been reported to appear relatively normal (14), systematic large-scale phenotyping showed a strongly decreased life expectancy in patients with MCT8 deficiency, with a median survival of 35 years (15). Proxies of the severity of both the peripheral and neurocognitive phenotype (being underweight early in life (1-3 years of age) and absence of head control before the age of 1.5 years, respectively) were strongly associated to increased mortality risk at a young age, with approximately 50% of severely affected patients dying in childhood. Importantly, sudden death was reported as a common cause of death. The observed high prevalence of cardiovascular abnormalities may point to a cardiac origin. Key characteristics such as cardiovascular function and nutritional status had been rarely documented in literature. These characteristics are potential key determinants of early death. Such detailed quantitative natural history data may serve as a control cohort in the evaluation of future therapeutic interventions, with placebo-controlled studies deemed not feasible due to the rarity of MCT8 deficiency.

## Development of (Potential) Therapies

Currently, no therapeutic strategies are registered for MCT8 deficiency. The severity of this syndrome and its impact on quality of life of patients and their relatives, and the high mortality rate in infancy, warrant the need for adequate therapies. Since the description of the first patients with MCT8 deficiency, major research initiatives have been taken to design and develop therapies. This paragraph discusses the different treatment strategies that have been evaluated over time.

The ideal therapy should ameliorate or prevent the neurocognitive phenotype and should alleviate the peripheral thyrotoxicosis. It is generally accepted that putative beneficial effects on the neurocognitive phenotype are most prominent when treatment is commenced early in life, and are likely limited in older patients (analogous to congenital hypothyroidism). With symptoms of the peripheral phenotype linked to increased mortality risk at young age (15), therapies that can effectively and safely modulate the peripheral thyrotoxicosis should also be considered relevant, irrespective of their effects on neurocognitive outcomes.

Following the identification of the first patients with MCT8 deficiency and with hypothyroxinaemia being observed, patients were initially treated with oral T4 supplementation [(8, 9, 64–82), reviewed in (18)]. Upon treatment, the already elevated serum T3 concentrations further increased in a subset of patients, as a consequence of conversion of T4 to T3 in peripheral tissues. In line with this increase in serum T3 concentrations, body weight and other markers of thyrotoxicosis even further deteriorated. Hence, treatment with T4 monotherapy appears to aggravate the peripheral thyrotoxic phenotype. As T4 is not able to enter the human brain without functional MCT8, no improvement of neurocognitive symptoms was observed. Therefore, it is generally accepted that postnatal treatment with T4 monotherapy is not recommended in MCT8 deficiency. Following these observations, it was hypothesized that by combining T4 with propylthiouracil (PTU), which inhibits DIO1 in peripheral tissues, the aggravation of the thyrotoxicosis could be overcome. This strategy has been reported in five patients [(55, 83–86), reviewed in (18)] and resulted in a decrease in serum T3 concentration and improvement of biochemical markers and clinical symptoms of the peripheral thyrotoxicosis (decrease in serum SHBG concentrations, increase in body weight, decrease in heart rate). Following the experiences with T4 monotherapy, no alterations in the neurocognitive phenotype were observed upon treatment. Interestingly, neurocognitive improvement was observed upon intra-uterine instillation of T4 in one mother pregnant of a foetus with MCT8 deficiency, followed by T4 + PTU combination therapy after birth (87). It remains unclear why prenatal treatment had a beneficial effect on neurocognition, whereas this is not the case for postnatal treatment (9, 78). This can possibly be explained by the timing of the intervention or differential expression of alternative thyroid hormone transporters in the prenatal brain. However, as PTU carries a high risk of severe adverse reactions (e.g. agranulocytosis and liver failure), the pros and cons of treatment with a combination of T4 and PTU should be carefully balanced, in particular in vulnerable patients such as patients with MCT8 deficiency (88). Recent studies found that intranasal thyroid hormone administration, theoretically bypassing the BBB, did not result in alterations in cerebral thyroid hormone content, but rather aggravated the peripheral thyrotoxicosis in Mct8 KO mice (89). Hence, following the conclusion of the authors, intranasal thyroid hormone administration is likely not a valid treatment option in patients with MCT8 deficiency. The efficacy of this therapy has not been formally tested in patients.

With classic (anti-)thyroid drugs not being effective in MCT8 deficiency, alternative treatment options were explored. Research initiatives have been focussing on compounds called thyroid hormone analogues, which are able to cross the cell membrane independent of MCT8, with intracellular effects and degradation similar to T3 (90). In Mct8 KO mice, the thyroid hormone analogue diiodothyropropionic acid (DITPA) was able to effectively normalize serum TSH concentrations and Dio1 expression in the liver, indicative of a decrease of the peripheral thyrotoxic state (91, 92). However, as Mct8 KO mice do not fully resemble the human cerebral pathophysiology of MCT8 deficiency, these studies were less suited to address the effect of DITPA on the neurocognitive phenotype. Following these pre-clinical studies, four patients (median age 25 months, range 8.5 – 25 months) were treated with DITPA on compassionate use basis for a median time of 38.5 months (range 26 – 40 months) (85). Upon treatment, serum T3 concentrations normalized and subsequent improvement of some markers of peripheral thyrotoxicosis, including body weight, was observed in some of the subjects. However, DITPA treatment did not result in improvements of the neurocognitive phenotypes in these patients, despite their relatively young age. After these reports, additional work showed that DITPA is able to cross the murine placenta independent of Mct8 (93). If similar observations are made for the human situation, this would classify DITPA as a potentially suitable compound for prenatal treatment of foetuses with MCT8 deficiency; this, however, remains to be elucidated. In zebrafish larvae lacking Mct8, DITPA was able to partially restore myelination six days after fertilization (94). Moreover, Lee et al. showed that DITPA restores myelination in oligodendrocytes derived from human embryonic stem cells, in which MCT8 was knocked down using short hairpin (sh)RNA technique (95). It should be noted that, despite its beneficial effects on the peripheral phenotype, DITPA is currently not commercially available.

Alongside DITPA, the therapeutic potential of the thyroid hormone analogue triiodothyroacetic acid (Triac) for MCT8 deficiency has extensively been studied. Triac is a naturally occurring thyroid hormone metabolite, which potently binds and activates the TRs and enters the cell independent of MCT8 (22). Following these observations, the efficacy of Triac was evaluated in Mct8/Oatp1c1 DKO mice. Importantly, a clear improvement in brain development was observed when mice were treated directly after birth (from postnatal day 1 to 12; dose 50 ng/g body weight), with complete restoration when high doses of Triac (400 ng/g body weight) were administered, as illustrated by normalization of cerebellar Purkinje cell dendritogenesis (calbindin immunoreactivity) and myelination (MBP immunoreactivity) (22). In line with these findings, Triac was able to completely rescue myelination in *mct8^-/-^* zebrafish larvae six days after fertilization, when brain damage has already occurred (94). An international phase II trial including 46 patients with a median age of 7.1 years demonstrated that Triac effectively reduces the high serum T3 concentrations and improved subsequent clinical and biochemical features of thyrotoxicosis, including body weight, heart rate, occurrence of PACs, SHBG and creatinine. In a subset of patients, treatment was extended up until 3 years and resulted in sustained beneficial effects on the peripheral phenotype. Except transient signs of increased thyrotoxicosis in a small subset of patients, no (severe) adverse events related to Triac were observed. Furthermore, a trend of improvement in neurodevelopment was observed in patients treated early in life (younger than four years at baseline) (96). Previous studies have demonstrated transplacental passage of Triac in women, also making Triac a suitable compound for prenatal treatment of foetuses with MCT8 deficiency (97).

In pursuit of the identification of thyromimetic molecules with a larger bioavailability in the brain, the thyroid hormone analogue sobetirome and its prodrug sob-AM2 have been studied in Mct8/Dio2 DKO mice (98). Although upon treatment serum T3 concentrations decreased, the effects of sobetirome and sob-AM2 on the peripheral phenotype and its safety profile remain unclear. In particular, upon sobetirome and sob-AM2 treatment, expression of T3-responsive genes did not alter in the liver and were increased in the heart, suggesting that these compounds may aggravate the peripheral thyrotoxic state. Cerebral *Hairless* expression levels were restored, indicative of a beneficial effect on cerebral thyroid hormone signalling. Additional evaluation of efficacy and safety is warranted before this drug can be used in a clinical setting.

Next to recovering thyroid hormone signalling *via* thyroid hormone analogues that enter the cell independent of MCT8, attempts to recover MCT8 function have been made. Gene therapy using adeno-associated virus 9 (AAV9) vector containing human MCT8 cDNA was able to increase cerebral thyroid hormone signalling in Mct8 KO mice (99). These effects were only observed after intravenous administration, but not after intracerebroventricular administration, which underlines the relevance of MCT8 at the BBB. Using a similar approach, Zada et al. showed that upregulation of the fusion protein Mct8-tagRFP completely rescued hypomyelination in *mct8^-/-^* zebrafish larvae (94). The effects of these different forms of gene therapy on the peripheral thyrotoxic phenotype as well as their safety profiles are to be explored. Hence, clinical evaluation of these promising therapies is not yet applicable.

In addition, different chemical chaperones have been exploited to improve trafficking and stability of mutant MCT8, thus potentially increasing MCT8-mediated thyroid hormone transport across the cell membrane. Depending on the cell model, different effects have been found. In overexpression models of the p.F501del mutation, treatment with phenylbutyrate and dimethylsulfoxide enhanced residual MCT8 transport capacity (100, 101). In fibroblasts containing this mutation, considered the most representative *ex vivo* model, these effects were not observed (25). The effects of such molecules on the thyrotoxic and neurocognitive phenotype are unclear, as none of these compounds have been tested in animal models for MCT8 deficiency. As this potential therapy is only applicable for a subset of mutations, its role in clinical praxis will likely be limited.

## Conclusions and Future Perspectives

Since the first recognition of MCT8 deficiency, major discoveries have been made, helping to better understand this rare disorder. By utilizing different *in vitro, ex vivo* and *in vivo* models, it became clear that MCT8 particularly plays a crucial role at the BBB. Additional studies are warranted to fully understand the role of MCT8 within other parts of the brain. Results from recent deep-phenotyping re-emphasized the relevance of the peripheral phenotype in patients with MCT8 deficiency, as being underweight early in life as a proxy of the peripheral thyrotoxicosis, has been linked to increased mortality risk at a young age. These findings stress the importance of the thyrotoxic features as therapeutic target and thus the availability of treatment options that modulate the peripheral phenotype. The thyroid hormone analogue Triac, the only available safe treatment at this moment, is able to effectively and safely reduce the peripheral thyrotoxicosis in patients and might improve the neurocognitive phenotype when treatment is initiated early in life.

Currently, a second international phase IIb trial studies the effect of Triac on neurocognitive outcomes in young patients (<30 months at baseline; NCT02396459). Another research initiative aims to treat women pregnant of a foetus with MCT8 deficiency with intra-uterine instillation of DITPA on compassionate use basis, in order to evaluate its effects on brain development when commenced early in life (NCT04143295). Aiming to gain a deeper understanding of the needs of patients and their parents and physicians, an international registry linked to the European Reference Network on Rare Endocrine Conditions centralizes knowledge on MCT8 deficiency (https://mct8registry.erasmusmc.nl/en/index.html). Together with other studies, these initiatives aim to provide additional insights in MCT8 deficiency and to decipher whether thyroid hormone analogues are able to modulate the neurocognitive phenotype when treatment is initiated early in life. With the availability of promising (prenatal) therapy, efforts should be made to detect MCT8 deficiency as early in life as possible. This goal could potentially be achieved by redefining neonatal screening programs. Given the rarity and complexity of MCT8 deficiency, international collaboration amongst researchers is warranted to attain these goals.

## Author Contributions

FSvG, NG, SG, and WEV reviewed available literature and wrote the manuscript. All authors contributed to the article and approved the submitted version.

## Funding

The authors acknowledge funding support by the Sherman Foundation (WEV), Eurostars (E11337; WEV) and Erasmus MC fellowship (WEV).

## Conflict of Interest

The Erasmus Medical Centre (Rotterdam, Netherlands), which employs FSvG, NG, SG, and WEV receives royalties from Rare Thyroid Therapeutics (the manufacturer of Triac), dependent on commercialisation. None of the authors will benefit personally from any royalties. None of the authors have personal disclosures relevant to this work.

## Publisher’s Note

All claims expressed in this article are solely those of the authors and do not necessarily represent those of their affiliated organizations, or those of the publisher, the editors and the reviewers. Any product that may be evaluated in this article, or claim that may be made by its manufacturer, is not guaranteed or endorsed by the publisher.

## References

[1] Bernal J Guadano-Ferraz A Morte B. Thyroid Hormone Transporters–Functions and Clinical Implications. Nat Rev Endocrinol (2015) 11(7):406–17. 10.1038/nrendo.2015.66 25942657

[2] Mullur R Liu YY Brent GA. Thyroid Hormone Regulation of Metabolism. Physiol Rev (2014) 94(2):355–82. 10.1152/physrev.00030.2013 PMC4044302 24692351

[3] Yen PM. Physiological and Molecular Basis of Thyroid Hormone Action. Physiol Rev (2001) 81(3):1097–142. 10.1152/physrev.2001.81.3.1097 11427693

[4] Bianco AC Dumitrescu A Gereben B Ribeiro MO Fonseca TL Fernandes GW. Paradigms of Dynamic Control of Thyroid Hormone Signaling. Endocr Rev (2019) 40(4):1000–47. 10.1210/er.2018-00275 PMC6596318 31033998

[5] Bochukova E Schoenmakers N Agostini M Schoenmakers E Rajanayagam O Keogh JM. A Mutation in the Thyroid Hormone Receptor Alpha Gene. N Engl J Med (2012) 366(3):243–9. 10.1056/NEJMoa1110296 22168587

[6] van Mullem A van Heerebeek R Chrysis D Visser E Medici M Andrikoula M. Clinical Phenotype and Mutant Tralpha1. N Engl J Med (2012) 366(15):1451–3. 10.1056/NEJMc1113940 22494134

[7] Refetoff S DeWind LT DeGroot LJ. Familial Syndrome Combining Deaf-Mutism, Stuppled Epiphyses, Goiter and Abnormally High PBI: Possible Target Organ Refractoriness to Thyroid Hormone. J Clin Endocrinol Metab (1967) 27(2):279–94. 10.1210/jcem-27-2-279 4163616

[8] Friesema EC Grueters A Biebermann H Krude H von Moers A Reeser M. Association Between Mutations in a Thyroid Hormone Transporter and Severe X-Linked Psychomotor Retardation. Lancet (2004) 364(9443):1435–7. 10.1016/S0140-6736(04)17226-7 15488219

[9] Dumitrescu AM Liao XH Best TB Brockmann K Refetoff S. A Novel Syndrome Combining Thyroid and Neurological Abnormalities Is Associated With Mutations in a Monocarboxylate Transporter Gene. Am J Hum Genet (2004) 74(1):168–75. 10.1086/380999 PMC1181904 14661163

[10] Stromme P Groeneweg S Lima de Souza EC Zevenbergen C Torgersbraten A Holmgren A. Mutated Thyroid Hormone Transporter OATP1C1 Associates With Severe Brain Hypometabolism and Juvenile Neurodegeneration. Thyroid (2018) 28(11):1406–15. 10.1089/thy.2018.0595 30296914

[11] Dumitrescu AM Liao XH Abdullah MS Lado-Abeal J Majed FA Moeller LC. Mutations in SECISBP2 Result in Abnormal Thyroid Hormone Metabolism. Nat Genet (2005) 37(11):1247–52. 10.1038/ng1654 16228000

[12] Franca MM German A Fernandes GW Liao XH Bianco AC Refetoff S. Human Type 1 Iodothyronine Deiodinase (Dio1) Mutations Cause Abnormal Thyroid Hormone Metabolism. Thyroid (2021) 31(2):202–7. 10.1089/thy.2020.0253 PMC7891200 32718224

[13] Friesema EC Ganguly S Abdalla A Manning Fox JE Halestrap AP Visser TJ. Identification of Monocarboxylate Transporter 8 as a Specific Thyroid Hormone Transporter. J Biol Chem (2003) 278(41):40128–35. 10.1074/jbc.M300909200 12871948

[14] Schwartz CE May MM Carpenter NJ Rogers RC Martin J Bialer MG. Allan-Herndon-Dudley Syndrome and the Monocarboxylate Transporter 8 (MCT8) Gene. Am J Hum Genet (2005) 77(1):41–53. 10.1086/431313 15889350 PMC1226193

[15] Groeneweg S van Geest FS Abaci A Alcantud A Ambegaonkar GP Armour CM. Disease Characteristics of MCT8 Deficiency: An International, Retrospective, Multicentre Cohort Study. Lancet Diabetes Endocrinol (2020) 8(7):594–605. 10.1016/S2213-8587(20)30153-4 32559475 PMC7611932

[16] Zwanziger D Schmidt M Fischer J Kleinau G Braun D Schweizer U. The Long N-Terminus of the Human Monocarboxylate Transporter 8 Is a Target of Ubiquitin-Dependent Proteasomal Degradation Which Regulates Protein Expression and Oligomerization Capacity. Mol Cell Endocrinol (2016) 434:278–87. 10.1016/j.mce.2016.05.017 27222294

[17] Groeneweg S van den Berge A Lima de Souza EC Meima ME Peeters RP Visser WE. Insights Into the Mechanism of MCT8 Oligomerization. J Endocr Soc (2020) 4(8):bvaa080. 10.1210/jendso/bvaa080 32724870 PMC7375341

[18] Groeneweg S van Geest FS Peeters RP Heuer H Visser WE. Thyroid Hormone Transporters. Endocr Rev (2020) 41(2):bnz008. 10.1210/endrev/bnz008 31754699

[19] Braun D Schweizer U. Thyroid Hormone Transport and Transporters. Vitam Horm (2018) 106:19–44. 10.1016/bs.vh.2017.04.005 29407435

[20] Groeneweg S Kersseboom S van den Berge A Dolcetta-Capuzzo A van Geest FS van Heerebeek REA. *In Vitro* Characterization of Human, Mouse, and Zebrafish MCT8 Orthologues. Thyroid (2019) 29(10):1499–510. 10.1089/thy.2019.0009 31436139

[21] Friesema EC Jansen J Jachtenberg JW Visser WE Kester MH Visser TJ. Effective Cellular Uptake and Efflux of Thyroid Hormone by Human Monocarboxylate Transporter 10. Mol Endocrinol (2008) 22(6):1357–69. 10.1210/me.2007-0112 PMC5419535 18337592

[22] Kersseboom S Horn S Visser WE Chen J Friesema EC Vaurs-Barriere C. *In Vitro* and Mouse Studies Supporting Therapeutic Utility of Triiodothyroacetic Acid in MCT8 Deficiency. Mol Endocrinol (2014) 28(12):1961–70. 10.1210/me.2014-1135 PMC5414784 25389909

[23] Horn S Kersseboom S Mayerl S Muller J Groba C Trajkovic-Arsic M. Tetrac can Replace Thyroid Hormone During Brain Development in Mouse Mutants Deficient in the Thyroid Hormone Transporter Mct8. Endocrinology (2013) 154(2):968–79. 10.1210/en.2012-1628 23307789

[24] Johannes J Jayarama-Naidu R Meyer F Wirth EK Schweizer U Schomburg L. Silychristin, A Flavonolignan Derived From the Milk Thistle, Is a Potent Inhibitor of the Thyroid Hormone Transporter MCT8. Endocrinology (2016) 157(4):1694–701. 10.1210/en.2015-1933 26910310

[25] Groeneweg S van den Berge A Meima ME Peeters RP Visser TJ Visser WE. Effects of Chemical Chaperones on Thyroid Hormone Transport by MCT8 Mutants in Patient-Derived Fibroblasts. Endocrinology (2018) 159(3):1290–302. 10.1210/en.2017-00846 29309566

[26] Jomura R Akanuma SI Bauer B Yoshida Y Kubo Y Hosoya KI. Participation of Monocarboxylate Transporter 8, But Not P-Glycoprotein, in Carrier-Mediated Cerebral Elimination of Phenytoin Across the Blood-Brain Barrier. Pharm Res (2021) 38(1):113–25. 10.1007/s11095-021-03003-1 PMC8906351 33527223

[27] Nishimura M Naito S. Tissue-Specific Mrna Expression Profiles of Human Solute Carrier Transporter Superfamilies. Drug Metab Pharmacokinet (2008) 23(1):22–44. 10.2133/dmpk.23.22 18305372

[28] Alkemade A Friesema EC Unmehopa UA Fabriek BO Kuiper GG Leonard JL. Neuroanatomical Pathways for Thyroid Hormone Feedback in the Human Hypothalamus. J Clin Endocrinol Metab (2005) 90(7):4322–34. 10.1210/jc.2004-2567 15840737

[29] Vatine GD Shelest O Barriga BK Ofan R Rabinski T Mattis VB. Oligodendrocyte Progenitor Cell Maturation Is Dependent on Dual Function of MCT8 in the Transport of Thyroid Hormone Across Brain Barriers and the Plasma Membrane. Glia (2021) 69(9):2146–59. 10.1002/glia.24014 33956384

[30] Fliers E Alkemade A Wiersinga WM Swaab DF. Hypothalamic Thyroid Hormone Feedback in Health and Disease. Prog Brain Res (2006) 153:189–207. 10.1016/S0079-6123(06)53011-0 16876576

[31] Lopez-Espindola D Garcia-Aldea A Gomez de la Riva I Rodriguez-Garcia AM Salvatore D Visser TJ. Thyroid Hormone Availability in the Human Fetal Brain: Novel Entry Pathways and Role of Radial Glia. Brain Struct Funct (2019) 224(6):2103–19. 10.1007/s00429-019-01896-8 31165302

[32] Wilpert NM Krueger M Opitz R Sebinger D Paisdzior S Mages B. Spatiotemporal Changes of Cerebral Monocarboxylate Transporter 8 Expression. Thyroid (2020) 30(9):1366–83. 10.1089/thy.2019.0544 32143555

[33] Leitch VD Di Cosmo C Liao XH O’Boy S Galliford TM Evans H. An Essential Physiological Role for MCT8 in Bone in Male Mice. Endocrinology (2017) 158(9):3055–66. 10.1210/en.2017-00399 PMC5659673 28637283

[34] Di Cosmo C Liao XH Ye H Ferrara AM Weiss RE Refetoff S. Mct8-Deficient Mice Have Increased Energy Expenditure and Reduced Fat Mass That Is Abrogated by Normalization of Serum T3 Levels. Endocrinology (2013) 154(12):4885–95. 10.1210/en.2013-1150 PMC3836073 24029243

[35] Trajkovic-Arsic M Muller J Darras VM Groba C Lee S Weih D. Impact of Monocarboxylate Transporter-8 Deficiency on the Hypothalamus-Pituitary-Thyroid Axis in Mice. Endocrinology (2010) 151(10):5053–62. 10.1210/en.2010-0593 20702572

[36] Di Cosmo C Liao XH Dumitrescu AM Philp NJ Weiss RE Refetoff S. Mice Deficient in MCT8 Reveal a Mechanism Regulating Thyroid Hormone Secretion. J Clin Invest (2010) 120(9):3377–88. 10.1172/JCI42113 PMC2929715 20679730

[37] Friesema EC Jansen J Heuer H Trajkovic M Bauer K Visser TJ. Mechanisms of Disease: Psychomotor Retardation and High T3 Levels Caused by Mutations in Monocarboxylate Transporter 8. Nat Clin Pract Endocrinol Metab (2006) 2(9):512–23. 10.1038/ncpendmet0262 16957765

[38] Frints SG Lenzner S Bauters M Jensen LR Van Esch H des Portes V. MCT8 Mutation Analysis and Identification of the First Female With Allan-Herndon-Dudley Syndrome Due to Loss of MCT8 Expression. Eur J Hum Genet (2008) 16(9):1029–37. 10.1038/ejhg.2008.66 18398436

[39] Dumitrescu AM Liao XH Weiss RE Millen K Refetoff S. Tissue-Specific Thyroid Hormone Deprivation and Excess in Monocarboxylate Transporter (Mct) 8-Deficient Mice. Endocrinology (2006) 147(9):4036–43. 10.1210/en.2006-0390 16709608

[40] Trajkovic M Visser TJ Mittag J Horn S Lukas J Darras VM. Abnormal Thyroid Hormone Metabolism in Mice Lacking the Monocarboxylate Transporter 8. J Clin Invest (2007) 117(3):627–35. 10.1172/JCI28253 PMC1797602 17318265

[41] Mayerl S Muller J Bauer R Richert S Kassmann CM Darras VM. Transporters MCT8 and OATP1C1 Maintain Murine Brain Thyroid Hormone Homeostasis. J Clin Invest (2014) 124(5):1987–99. 10.1172/JCI70324 PMC4001533 24691440

[42] Visser WE Heuer H Visser TJ. Triiodothyroacetic Acid Treatment in MCT8 Deficiency: A Word of Nuance. Thyroid (2016) 26(5):615–7. 10.1089/thy.2016.0191 27042766

[43] Barez-Lopez S Grijota-Martinez C Auso E Fernandez-de Frutos M Montero-Pedrazuela A Guadano-Ferraz A. Adult Mice Lacking Mct8 and Dio2 Proteins Present Alterations in Peripheral Thyroid Hormone Levels and Severe Brain and Motor Skill Impairments. Thyroid (2019) 29(11):1669–82. 10.1089/thy.2019.0068 31359845

[44] Vatine GD Zada D Lerer-Goldshtein T Tovin A Malkinson G Yaniv K. Zebrafish as a Model for Monocarboxyl Transporter 8-Deficiency. J Biol Chem (2013) 288(1):169–80. 10.1074/jbc.M112.413831 PMC3537011 23161551

[45] Vancamp P Deprez MA Remmerie M Darras VM. Deficiency of the Thyroid Hormone Transporter Monocarboxylate Transporter 8 in Neural Progenitors Impairs Cellular Processes Crucial for Early Corticogenesis. J Neurosci (2017) 37(48):11616–31. 10.1523/JNEUROSCI.1917-17.2017 PMC6705747 29109240

[46] Vatine GD Al-Ahmad A Barriga BK Svendsen S Salim A Garcia L. Modeling Psychomotor Retardation Using Ipscs From MCT8-Deficient Patients Indicates a Prominent Role for the Blood-Brain Barrier. Cell Stem Cell (2017) 20(6):831–43.e5. 10.1016/j.stem.2017.04.002 28526555 PMC6659720

[47] Mayerl S Heuer H Ffrench-Constant C. Hippocampal Neurogenesis Requires Cell-Autonomous Thyroid Hormone Signaling. Stem Cell Rep (2020) 14(5):845–60. 10.1016/j.stemcr.2020.03.014 PMC7220957 32302557

[48] Lopez-Espindola D Morales-Bastos C Grijota-Martinez C Liao XH Lev D Sugo E. Mutations of the Thyroid Hormone Transporter MCT8 Cause Prenatal Brain Damage and Persistent Hypomyelination. J Clin Endocrinol Metab (2014) 99(12):E2799–804. 10.1210/jc.2014-2162 PMC4255116 25222753

[49] Fliers E Unmehopa UA Alkemade A. Functional Neuroanatomy of Thyroid Hormone Feedback in the Human Hypothalamus and Pituitary Gland. Mol Cell Endocrinol (2006) 251(1-2):1–8. 10.1016/j.mce.2006.03.042 16707210

[50] Bianco AC Salvatore D Gereben B Berry MJ Larsen PR. Biochemistry, Cellular and Molecular Biology, and Physiological Roles of the Iodothyronine Selenodeiodinases. Endocr Rev (2002) 23(1):38–89. 10.1210/edrv.23.1.0455 11844744

[51] Liao XH Di Cosmo C Dumitrescu AM Hernandez A Van Sande J St Germain DL. Distinct Roles of Deiodinases on the Phenotype of Mct8 Defect: A Comparison of Eight Different Mouse Genotypes. Endocrinology (2011) 152(3):1180–91. 10.1210/en.2010-0900 PMC3040057 21285310

[52] Wirth EK Rijntjes E Meyer F Kohrle J Schweizer U. High T3, Low T4 Serum Levels in Mct8 Deficiency Are Not Caused by Increased Hepatic Conversion Through Type I Deiodinase. Eur Thyroid J (2015) 4(Suppl 1):87–91. 10.1159/000381021 26601078 PMC4640264

[53] Allan W Herndon CN Dudley FC. Some Examples of the Inheritance of Mental Deficiency: Apparently Sex-Linked Idiocy and Microcephaly. Am J Ment Defic (1944) 48:325–34.

[54] Fu J Korwutthikulrangsri M Ramos-Platt L Pierson TM Liao XH Refetoff S. Sorting Variants of Unknown Significance Identified by Whole Exome Sequencing: Genetic and Laboratory Investigations of Two Novel MCT8 Variants. Thyroid (2020) 30(3):463–5. 10.1089/thy.2018.0703 PMC7074892 31856685

[55] Visser WE Vrijmoeth P Visser FE Arts WF van Toor H Visser TJ. Identification, Functional Analysis, Prevalence and Treatment of Monocarboxylate Transporter 8 (MCT8) Mutations in a Cohort of Adult Patients With Mental Retardation. Clin Endocrinol (Oxf) (2013) 78(2):310–5. 10.1111/cen.12023 22924588

[56] Jansen J Friesema EC Kester MH Schwartz CE Visser TJ. Genotype-Phenotype Relationship in Patients With Mutations in Thyroid Hormone Transporter MCT8. Endocrinology (2008) 149(5):2184–90. 10.1210/en.2007-1475 PMC2734492 18187543

[57] Visser WE Jansen J Friesema EC Kester MH Mancilla E Lundgren J. Novel Pathogenic Mechanism Suggested by *Ex Vivo* Analysis of MCT8 (SLC16A2) Mutations. Hum Mutat (2009) 30(1):29–38. 10.1002/humu.20808 18636565

[58] Capri Y Friesema EC Kersseboom S Touraine R Monnier A Eymard-Pierre E. Relevance of Different Cellular Models in Determining the Effects of Mutations on SLC16A2/MCT8 Thyroid Hormone Transporter Function and Genotype-Phenotype Correlation. Hum Mutat (2013) 34(7):1018–25. 10.1002/humu.22331 23568789

[59] van Geest FS Meima ME Stuurman KE Wolf NI van der Knaap MS Lorea CF. Clinical and Functional Consequences of C-Terminal Variants in MCT8: A Case Series. J Clin Endocrinol Metab (2021) 106(2):539–53. 10.1210/clinem/dgaa795 PMC7823235 33141165

[60] Remerand G Boespflug-Tanguy O Tonduti D Touraine R Rodriguez D Curie A. Expanding the Phenotypic Spectrum of Allan-Herndon-Dudley Syndrome in Patients With SLC16A2 Mutations. Dev Med Child Neurol (2019) 61(12):1439–47. 10.1111/dmcn.14332 31410843

[61] Matheus MG Lehman RK Bonilha L Holden KR. Redefining the Pediatric Phenotype of X-Linked Monocarboxylate Transporter 8 (MCT8) Deficiency: Implications for Diagnosis and Therapies. J Child Neurol (2015) 30(12):1664–8. 10.1177/0883073815578524 25900139

[62] Vancamp P Demeneix BA Remaud S. Monocarboxylate Transporter 8 Deficiency: Delayed or Permanent Hypomyelination? Front Endocrinol (Lausanne) (2020) 11:283. 10.3389/fendo.2020.00283 32477268 PMC7237703

[63] Iwayama H Kakita H Iwasa M Adachi S Takano K Kikuchi M. Measurement of Reverse T3 Level and the T3 to Reverse T3 Ratio in Dried Blood Spot Samples at Birth may Facilitate Early Detection of Monocarboxylate Transporter 8 Deficiency. Thyroid (2021). 10.1210/jendso/bvab048.1998 PMC8558056 34049438

[64] Biebermann H Ambrugger P Tarnow P von Moers A Schweizer U Grueters A. Extended Clinical Phenotype, Endocrine Investigations and Functional Studies of a Loss-of-Function Mutation A150V in the Thyroid Hormone Specific Transporter MCT8. Eur J Endocrinol (2005) 153(3):359–66. 10.1530/eje.1.01980 16131597

[65] Choi JH Cho JH Kim JH Yoo EG Kim GH Yoo HW. Variable Clinical Characteristics and Molecular Spectrum of Patients With Syndromes of Reduced Sensitivity to Thyroid Hormone: Genetic Defects in the THRB and SLC16A2 Genes. Horm Res Paediatr (2018) 90(5):283–90. 10.1159/000493468 30497070

[66] Kim JH Kim YM Yum MS Choi JH Lee BH Kim GH. Clinical and Endocrine Features of Two Allan-Herndon-Dudley Syndrome Patients With Monocarboxylate Transporter 8 Mutations. Horm Res Paediatr (2015) 83(4):288–92. 10.1159/000371466 25896225

[67] Shimojima K Maruyama K Kikuchi M Imai A Inoue K Yamamoto T. Novel SLC16A2 Mutations in Patients With Allan-Herndon-Dudley Syndrome. Intractable Rare Dis Res (2016) 5(3):214–7. 10.5582/irdr.2016.01051 PMC4995413 27672545

[68] Fuchs O Pfarr N Pohlenz J Schmidt H. Elevated Serum Triiodothyronine and Intellectual and Motor Disability With Paroxysmal Dyskinesia Caused by a Monocarboxylate Transporter 8 Gene Mutation. Dev Med Child Neurol (2009) 51(3):240–4. 10.1111/j.1469-8749.2008.03125.x 19018842

[69] Novara F Groeneweg S Freri E Estienne M Reho P Matricardi S. Clinical and Molecular Characteristics of SLC16A2 (MCT8) Mutations in Three Families With the Allan-Herndon-Dudley Syndrome. Hum Mutat (2017) 38(3):260–4. 10.1002/humu.23140 27805744

[70] Ngu L. Developmental Delay and Abnormal Thyroid Function Test in Two Siblings Caused by a Novel Mutation in SLC16A2 Gene Affecting a Thyroid Hormone Specific Transporter (MCT8): First Report From Malaysia. In: 35th Malaysian Paediatrics Association Congress. Kuantan, Pahang, Malaysia, 27–30 June (2013).

[71] Philips AK Siren A Avela K Somer M Peippo M Ahvenainen M. X-Exome Sequencing in Finnish Families With Intellectual Disability–Four Novel Mutations and Two Novel Syndromic Phenotypes. Orphanet J Rare Dis (2014) 9:49. 10.1186/1750-1172-9-49 24721225 PMC4022384

[72] Anik A Kersseboom S Demir K Catli G Yis U Bober E. Psychomotor Retardation Caused by a Defective Thyroid Hormone Transporter: Report of Two Families With Different MCT8 Mutations. Horm Res Paediatr (2014) 82(4):261–71. 10.1159/000365191 25247785

[73] Ugrasbul FA HH. A Patient Presenting With Central Hypothyroidism, Developmental Delay and Poor Head Control. Should We Be Checking T3 Levels? Horm Res (2009) 72(Suppl 1):458–9.

[74] Papadimitriou A Dumitrescu AM Papavasiliou A Fretzayas A Nicolaidou P Refetoff S. A Novel Monocarboxylate Transporter 8 Gene Mutation as a Cause of Severe Neonatal Hypotonia and Developmental Delay. Pediatrics (2008) 121(1):e199–202. 10.1542/peds.2007-1247 18166539

[75] Kakinuma H Itoh M Takahashi H. A Novel Mutation in the Monocarboxylate Transporter 8 Gene in a Boy With Putamen Lesions and Low Free T4 Levels in Cerebrospinal Fluid. J Pediatr (2005) 147(4):552–4. 10.1016/j.jpeds.2005.05.012 16227048

[76] Levenson A Tan WH Huang SA. Diagnostic Dilemma: A 3-Year Old Boy With Global Developmental Delay, Truncal Hypotonia, Peripheral Hypertonia, and Central Hypothyroidism. In: 95th Annual Meeting of the Endocrine Society. San Francisco, CA, June 15–18 (2013).

[77] Herzovich V Vaiani E Marino R Dratler G Lazzati JM Tilitzky S. Unexpected Peripheral Markers of Thyroid Function in a Patient With a Novel Mutation of the MCT8 Thyroid Hormone Transporter Gene. Horm Res (2007) 67(1):1–6. 10.1159/000095805 16974106

[78] Crushell E Reardon W. Elevated TSH Levels in a Mentally Retarded Boy. Eur J Pediatr (2010) 169(5):573–5. 10.1007/s00431-009-1075-0 19936787

[79] Namba N Etani Y Kitaoka T Nakamoto Y Nakacho M Bessho K. Clinical Phenotype and Endocrinological Investigations in a Patient With a Mutation in the MCT8 Thyroid Hormone Transporter. Eur J Pediatr (2008) 167(7):785–91. 10.1007/s00431-007-0589-6 17899191

[80] Garcia-de Teresa B Gonzalez-Del Angel A Reyna-Fabian ME Ruiz-Reyes Mde L Calzada-Leon R Perez-Enriquez B. Deletion of Exon 1 of the SLC16A2 Gene: A Common Occurrence in Patients With Allan-Herndon-Dudley Syndrome. Thyroid (2015) 25(3):361–7. 10.1089/thy.2014.0284 25517855

[81] Zung A Visser TJ Uitterlinden AG Rivadeneira F Friesema EC. A Child With a Deletion in the Monocarboxylate Transporter 8 Gene: 7-Year Follow-Up and Effects of Thyroid Hormone Treatment. Eur J Endocrinol (2011) 165(5):823–30. 10.1530/EJE-11-0358 21896621

[82] Filho HC Marui S Manna TD Brust ES Radonsky V Kuperman H. Novel Mutation in MCT8 Gene in a Brazilian Boy With Thyroid Hormone Resistance and Severe Neurologic Abnormalities. Arq Bras Endocrinol Metabol (2011) 55(1):60–6. 10.1590/S0004-27302011000100008 21468521

[83] Wemeau JL Pigeyre M Proust-Lemoine E d’Herbomez M Gottrand F Jansen J. Beneficial Effects of Propylthiouracil Plus L-Thyroxine Treatment in a Patient With a Mutation in MCT8. J Clin Endocrinol Metab (2008) 93(6):2084–8. 10.1210/jc.2007-2719 18334584

[84] Gika AD Siddiqui A Hulse AJ Edward S Fallon P McEntagart ME. White Matter Abnormalities and Dystonic Motor Disorder Associated With Mutations in the SLC16A2 Gene. Dev Med Child Neurol (2010) 52(5):475–82. 10.1111/j.1469-8749.2009.03471.x PMC5800746 19811520

[85] Verge CF Konrad D Cohen M Di Cosmo C Dumitrescu AM Marcinkowski T. Diiodothyropropionic Acid (DITPA) in the Treatment of MCT8 Deficiency. J Clin Endocrinol Metab (2012) 97(12):4515–23. 10.1210/jc.2012-2556 PMC3513545 22993035

[86] Fu J Refetoff S Dumitrescu AM. Inherited Defects of Thyroid Hormone-Cell-Membrane Transport: Review of Recent Findings. Curr Opin Endocrinol Diabetes Obes (2013) 20(5):434–40. 10.1097/01.med.0000432531.03233.ad PMC4061907 23974772

[87] Refetoff S Pappa T Williams MK Matheus MG Liao XH Hansen K. Prenatal Treatment of Thyroid Hormone Cell Membrane Transport Defect Caused by MCT8 Gene Mutation. Thyroid (2020) 31(5):713–20. 10.1089/thy.2020.0306 PMC8110025 32746752

[88] Rivkees SA Mattison DR. Ending Propylthiouracil-Induced Liver Failure in Children. N Engl J Med (2009) 360(15):1574–5. 10.1056/NEJMc0809750 19357418

[89] Grijota-Martinez C Barez-Lopez S Auso E Refetoff S Frey WH 2nd Guadano-Ferraz A. Intranasal Delivery of Thyroid Hormones in MCT8 Deficiency. PloS One (2020) 15(7):e0236113. 10.1371/journal.pone.0236113 32687511 PMC7371167

[90] Groeneweg S Peeters RP Visser TJ Visser WE. Therapeutic Applications of Thyroid Hormone Analogues in Resistance to Thyroid Hormone (RTH) Syndromes. Mol Cell Endocrinol (2017) 458:82–90. 10.1016/j.mce.2017.02.029 28235578

[91] Di Cosmo C Liao XH Dumitrescu AM Weiss RE Refetoff S. A Thyroid Hormone Analog With Reduced Dependence on the Monocarboxylate Transporter 8 for Tissue Transport. Endocrinology (2009) 150(9):4450–8. 10.1210/en.2009-0209 PMC2736078 19497976

[92] Ferrara AM Liao XH Ye H Weiss RE Dumitrescu AM Refetoff S. The Thyroid Hormone Analog DITPA Ameliorates Metabolic Parameters of Male Mice With Mct8 Deficiency. Endocrinology (2015) 156(11):3889–94. 10.1210/en.2015-1234 PMC4606752 26322373

[93] Ferrara AM Liao XH Gil-Ibanez P Bernal J Weiss RE Dumitrescu AM. Placenta Passage of the Thyroid Hormone Analog DITPA to Male Wild-Type and Mct8-Deficient Mice. Endocrinology (2014) 155(10):4088–93. 10.1210/en.2014-1085 PMC4164925 25051435

[94] Zada D Tovin A Lerer-Goldshtein T Appelbaum L. Pharmacological Treatment and BBB-Targeted Genetic Therapy for MCT8-Dependent Hypomyelination in Zebrafish. Dis Model Mech (2016) 9(11):1339–48. 10.1242/dmm.027227 PMC5117236 27664134

[95] Lee JY Kim MJ Deliyanti D Azari MF Rossello F Costin A. Overcoming Monocarboxylate Transporter 8 (MCT8)-Deficiency to Promote Human Oligodendrocyte Differentiation and Myelination. EBioMedicine (2017) 25:122–35. 10.1016/j.ebiom.2017.10.016 PMC5704066 29111262

[96] Groeneweg S Peeters RP Moran C Stoupa A Auriol F Tonduti D. Effectiveness and Safety of the Tri-Iodothyronine Analogue Triac in Children and Adults With MCT8 Deficiency: An International, Single-Arm, Open-Label, Phase 2 Trial. Lancet Diabetes Endocrinol (2019) 7(9):695–706. 10.1016/S2213-8587(19)30155-X 31377265 PMC7611958

[97] Cortelazzi D Morpurgo PS Zamperini P Fisher DA Beck-Peccoz P Wu SY. Maternal Compound W Serial Measurements for the Management of Fetal Hypothyroidsm. Eur J Endocrinol (1999) 141(6):570–8. 10.1530/eje.0.1410570 10601958

[98] Barez-Lopez S Hartley MD Grijota-Martinez C Scanlan TS Guadano-Ferraz A. Sobetirome and Its Amide Prodrug Sob-AM2 Exert Thyromimetic Actions in Mct8-Deficient Brain. Thyroid (2018) 28(9):1211–20. 10.1089/thy.2018.0008 PMC6154442 29845892

[99] Iwayama H Liao XH Braun L Barez-Lopez S Kaspar B Weiss RE. Adeno Associated Virus 9-Based Gene Therapy Delivers a Functional Monocarboxylate Transporter 8, Improving Thyroid Hormone Availability to the Brain of Mct8-Deficient Mice. Thyroid (2016) 26(9):1311–9. 10.1089/thy.2016.0060 PMC5036314 27432638

[100] Braun D Schweizer U. The Chemical Chaperone Phenylbutyrate Rescues MCT8 Mutations Associated With Milder Phenotypes in Patients With Allan-Herndon-Dudley Syndrome. Endocrinology (2017) 158(3):678–91. 10.1210/en.2016-1530 27977298

[101] Braun D Schweizer U. Efficient Activation of Pathogenic Deltaphe501 Mutation in Monocarboxylate Transporter 8 by Chemical and Pharmacological Chaperones. Endocrinology (2015) 156(12):4720–30. 10.1210/en.2015-1393 26368820

